# Numerical and Experimental Study of Laser Surface Modification Using a High-Power Fiber CW Laser

**DOI:** 10.3390/ma19020343

**Published:** 2026-01-15

**Authors:** Evaggelos Kaselouris, Alexandros Gosta, Efstathios Kamposos, Dionysios Rouchotas, George Vernardos, Helen Papadaki, Alexandros Skoulakis, Yannis Orphanos, Makis Bakarezos, Ioannis Fitilis, Nektarios A. Papadogiannis, Michael Tatarakis, Vasilis Dimitriou

**Affiliations:** 1 Institute of Plasma Physics and Lasers-IPPL, University Research and Innovation Centre, Hellenic Mediterranean University, 74150 Rethymnon, Greece; 2 Physical Acoustics and Optoacoustics Laboratory, Department of Music Technology and Acoustics, Hellenic Mediterranean University, 74133 Rethymnon, Greece; 3 Department of Electronic Engineering, Hellenic Mediterranean University, 73133 Chania, Greece

**Keywords:** laser surface treatment, fiber laser, finite element method, etching, engraving

## Abstract

This work presents a combined numerical and experimental investigation into the laser machining of aluminum alloy Al 1050 H14 using a high-power Continuous Wave (CW) fiber laser. Advanced three-dimensional, coupled thermal–structural Finite Element Method (FEM) simulations are developed to model key laser–material interaction processes, including laser-induced plastic deformation, laser etching, and engraving. Cases for both static single-shot and dynamic linear scanning laser beams are investigated. The developed numerical models incorporate a Gaussian heat source and the Johnson–Cook constitutive model to capture elastoplastic, damage, and thermal effects. The simulation results, which provide detailed insights into temperature gradients, displacement fields, and stress–strain evolution, are rigorously validated against experimental data. The experiments are conducted on an integrated setup comprising a 2 kW TRUMPF CW fiber laser hosted on a 3-axis CNC milling machine, with diagnostics including thermal imaging, thermocouples, white-light interferometry, and strain gauges. The strong agreement between simulations and measurements confirms the predictive capability of the developed FEM framework. Overall, this research establishes a reliable computational approach for optimizing laser parameters, such as power, dwell time, and scanning speed, to achieve precise control in metal surface treatment and modification applications.

## 1. Introduction

Lasers have revolutionized modern manufacturing, offering unparalleled precision, speed, and flexibility in material processing [1,2,3]. Among various laser types, high-power fiber lasers have emerged as a dominant technology for industrial applications due to their excellent beam quality, high efficiency, and robustness [4,5]. Continuous Wave (CW) fiber lasers enable high-speed processing and are extensively used for a wide range of operations [6,7,8,9,10,11,12,13,14,15] beyond simple cutting, including marking, engraving, etching (occurs when material is removed by less than 25 μm), and surface modification. These processes are critical in industries such as aerospace, automotive, and electronics, where permanent identification, functional surface structuring, and precision micromachining are required.

The effectiveness of laser material processing is governed by a complex interplay of parameters, including laser power, irradiation time, scanning speed, and material properties [16,17]. The intense, localized heating induces rapid thermal gradients, leading to thermal expansion, phase changes, and the development of residual stresses and plastic strain. A profound understanding of these thermomechanical phenomena is essential for optimizing process outcomes and avoiding defects. However, the transient and multi-physics nature of laser-material interactions makes purely experimental optimization costly and time-consuming.

Computational modeling, particularly FEM [18,19,20], has become an indispensable tool for explaining the underlying mechanisms of multiphysics processes. FEM enables the simulation of coupled thermal-structural responses [21,22,23,24,25,26,27,28,29,30], providing detailed spatial and temporal analyses of temperature, displacement, stress, and strain that are often challenging to measure experimentally with sufficient resolution. By validating these models with experimental data, a powerful predictive capability can be established, reducing the needs for extensive trial-and-error experimentation.

While significant research has been devoted to laser cutting [7,11,22,30] and welding [23,31,32,33,34], a comprehensive numerical and experimental study focusing specifically on non-ablative and moderate-ablative regimes—such as plastic deformation, etching and engraving—remains an open area of investigation. The work is focused on the aluminum alloy Al 1050 H14 and pursues three key objectives through an integrated approach: first, to develop high-fidelity 3D transient coupled thermo-structural FEM models for both static and dynamic laser irradiation; second, to rigorously validate the simulations against experimental data obtained from a high-power CW fiber laser integrated with a 3-axis CNC system and experimental diagnostics; and ultimately, to analyze the correlation between laser parameters and the thermomechanical material response, thereby demonstrating the predictive power of the numerical models for optimizing laser-based surface modification. Thus, the high power of the CW fiber laser is limited to the threshold value of 250 W, by exploiting its modulated power control capability. This approach enables a controlled investigation of surface modifications restricted to plastic deformation, etching, and shallow, high-precision engraving under both static single-shot and dynamic CW processing conditions. Therefore, the experimental investigation of high-precision surface imprinting and engraving, enables direct measurement and comparison with the FEM simulation results for validation.

The key novel aspects of the study include: (a) the development of a precise 3D transient coupled thermo-structural model that accurately simulates both static and dynamic laser irradiation, capturing the transition from plastic deformation to material removal (etching, engraving) within a single computational framework, (b) the extensive experimental validation using a multi-diagnostic approach (thermal imaging, interferometry, strain gauges) that provides simultaneous data on thermal gradients, surface topography, and mechanical strain, offering a holistic view for model verification, and (c) the demonstration of this model’s predictive capability for controlling surface modification outcomes—from permanent marking via plastic strain to precise engraving depth—by tuning laser parameters, providing a valuable tool for optimizing industrial laser machining processes without the need for costly trial-and-error.

## 2. Mathematical Modeling and Numerical Simulations

A multiphysics computational model is developed to simulate the transient, coupled thermo-mechanical phenomena during laser processing. This section outlines the governing equations, constitutive material models, and numerical implementation strategies employed in the FEM framework. Three-dimensional transient coupled thermal-structural numerical simulations were conducted using the LS-DYNA R15.0.2 software [35].

### 2.1. Mathematical Model

The thermal response is governed by the heat conduction equation, which accounts for heat transfer and a moving laser heat source:

(1) $$\rho \left(T\right)c_{p}\left(T\right)\left(\frac{\partial T\left(r,t\right)}{\partial t}+V_{x}\frac{\partial T\left(r,t\right)}{\partial x}\right)=\frac{\partial }{\partial r}\left(\kappa \left(Τ\right)\frac{\partial Τ\left(r,t\right)}{\partial r}\right)+Q(r,t)$$

where *r* = *x*, *y*, *z* the coordinates, ρ is the density, $c_{p}$ is the specific heat at constant pressure, T is the temperature, t is time, k is the thermal conductivity, $V_{x}$ is the laser scanning speed, and Q is the volumetric heat source, the absorbed laser energy per unit volume, per unit time by the sample. The motion of the laser beam is along the X-axis. The latent heat of melting is also considered when temperature exceeds the melting point.

The mechanical response to the induced thermal stresses is described by the equation of motion:

(2) $$\rho \left(T\right)\frac{\partial^{2}U(r,t)}{\partial t^{2}}=\mu \nabla^{2}U\left(r,T\right)+\left(\lambda +\mu \right)\nabla \left[\nabla U\left(r,t\right)\right]-a\left(3\lambda +2\mu \right)\nabla Τ(r,t)$$

where *U* represents the displacement, *α* the thermal expansion coefficient and *λ*, *μ* are Lame constants depending on the material. The mechanical behavior of the Al target can be expressed by the following Equations (3) and (4) where *σ_ij_* and *ε_ij_* are the stress and strain tensors in the *ij* plane, respectively, and *T*_0_ the ambient temperature:

(3) $$\sigma_{ij}=2\mu \varepsilon_{ij}+\lambda \varepsilon_{kk}\delta_{ij}-\left(3\lambda +2\mu \right)a(T-T_{0})\delta_{ij}$$

(4) $$\varepsilon_{ij}=\frac{1}{2}\left(\frac{\partial U_{i}}{\partial x_{j}}+\frac{\partial U_{j}}{\partial x_{i}}\right)$$

The laser beam is a Gaussian volumetric heat source. The absorbed laser power density distribution is given by [36]:

(5) $$Q\left(x,y,z,t\right)=\frac{{2P}_{tot}}{\pi r_{b}^{2}H}e^{-(\frac{{2(z}^{2}+{\left(x-V_{x}t\right)}^{2})}{r_{b}^{2}})}e^{-(\frac{y}{H})}$$

where $P_{tot}=\eta P_{inc}$ is the total absorbed power, with η being the average absorptivity and $P_{inc}$ the incident laser power, $r_{b}$ is the laser beam radius, and H is the max workpiece thickness. Although optical absorption in aluminum occurs predominantly near the surface, the volumetric Gaussian heat source with exponential decay is adopted as an effective representation of near-surface absorption combined with rapid thermal diffusion during CW laser irradiation. On the millisecond timescales relevant to this study, heat conduction redistributes the absorbed energy into the bulk material, making the use of an effective penetration depth a common and practical modeling approach in FEM simulations of CW laser processing [36]. Additionally, a sensitivity analysis varying the decay parameter *H* by ±10% has been performed, which showed that peak temperatures varied by less than 2% and the spatial distribution of stresses and strains remained qualitatively unchanged.

### 2.2. Numerical Model

**Figure 1:**
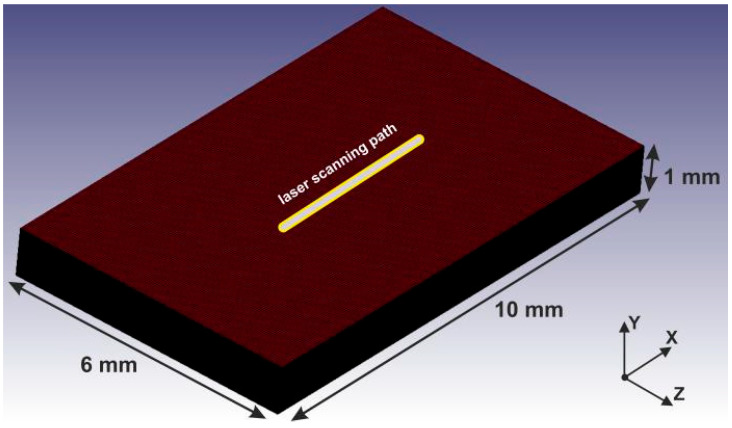
The discretized geometry of the model.

The Al 1050 domain, with dimensions 10 mm × 1 mm × 6 mm, is discretized using hexahedral elements of 50 μm × 10 μm × 50 μm, resulting in ~2.4 M solid elements and providing high spatial resolution in the laser interaction zone (see Figure 1). The bottom surface of the workpiece is constrained along the vertical direction according to the experimental conditions, while non-reflecting outflow boundary conditions were applied to the outer surfaces, except the top one where the laser heat source is applied. Free convection to the atmosphere is assumed to be present on the surfaces of the workpiece. The general boundary condition [22] is *q_fc_* = *h(T_s_* − *T*_0_*)*, where *h* the convective heat transfer coefficient of air (15 WK^−1^m^−2^), *T_s_* the surface temperature. Radiative heat losses were neglected in the present model, as preliminary simulations showed that radiation contributes less than 1% of the total heat loss even at the highest temperatures reached. Given the relatively short interaction times and the strong agreement between simulations and experimental thermal measurements, the inclusion of radiative losses was not expected to materially affect the predicted temperature evolution, in accordance with relevant laser machining studies [32,33,34]. Initially, the workpiece is assumed to be at constant ambient temperature. The simulations were executed on the ARIS high-performance computing (HPC) infrastructure [37], with a typical simulation time of 10 h for a 500 ms physical process duration.

A mesh convergence study was performed for the static irradiation case to ensure adequate resolution of the steep thermal gradients in the laser interaction zone (as shown in Table 1). Four mesh resolutions were evaluated, ranging from coarse to highly refined. The maximum temperature predicted at the laser spot converged toward approximately 570 °C, with deviations below 1% between the selected mesh (50 µm × 10 µm × 50 µm) and the finest mesh considered, which required approximately 50% higher computational cost. Further mesh refinement resulted in negligible changes in peak temperature while significantly increasing computational effort. Based on this analysis, the selected mesh was deemed sufficient to accurately capture the thermal response and was therefore used for all simulations presented in this study.

### 2.3. Material Properties and Laser Parameters

The aluminum alloy Al 1050 H14 [38] was selected for this study due to its status as a commercially pure, strain-hardened (H14 temper) material, which ensures highly uniform mechanical and thermal properties across its surface. This homogeneity is crucial for obtaining consistent and repeatable experimental data, thereby enabling a more reliable validation of the FEM simulations. Its well-documented properties, including high thermal conductivity and good formability, make it an ideal candidate for investigating the fundamental thermomechanical responses induced by laser processing.

The dynamic elastoplastic behavior of the workpiece is described by the Johnson-Cook constitutive strength material model [39], which accounts for the effects of plastic strain, strain rate, and temperature. The flow stress is expressed as:

(6) $$\sigma =(A+Β\varepsilon^{n})\left(1+Cln\frac{\dot{\varepsilon }}{\dot{\varepsilon_{0}}}\right)\left(1-(\frac{T-T_{0}}{T_{m}-T_{0}}{)}^{m}\right)$$

Parameters *A*, *B*, *C*, *n*, *m* are experimental constants which depend on the material and are determined from literature experimental results [40], while *T_m_* is the melting point of the material and $\dot{\varepsilon }$ and $\dot{\varepsilon }$*_0_* are the strain rate and the reference strain rate, respectively. The Johnson–Cook parameters adopted for Al 1050 H14 were obtained from literature sources and are applicable to moderate strain rates and elevated temperatures. Although CW laser processing involves steep thermal gradients, the associated strain rates remain within the validity range of the model, which is employed here as an engineering approximation for thermally driven plastic deformation rather than extreme dynamic loading. In case of high strain rates, the material model includes a fracture model that defines the equivalent plastic strain *ε_f_* in case of damage:

(7) $$\varepsilon_{f}=\left(D_{1}+D_{2}e^{D_{3}\frac{p}{\sigma_{VM}}}\right)\left(1+\frac{D_{4}ln\dot{\varepsilon }}{\dot{\varepsilon_{0}}}\right)\left(1+D_{5}\frac{T-T_{r}}{T_{m}-T_{r}}\right)$$

(8) $$D=\Sigma \left(\frac{\Delta \varepsilon }{\varepsilon_{f}}\right)$$

*D*_1_–*D*_5_ are the failure parameters of the material, while *D* is the damage parameter. When *D* becomes 1, the material fractures. Temperature dependent values of the thermal properties were also considered [41]. To model the volumetric response of the material under high-pressure wave propagation generated by rapid laser heating, the Gruneisen equation of state [42] was applied in LS-DYNA. The material properties and constitutive parameters employed in the numerical simulations are summarized in Table 2, Table 3 and Table 4.

Regarding the laser parameters, the CW laser beam diameter is 250 μm with the laser power ranging from 0.2 to 0.25 kW according to the experimental measurements and the laser speed ranges from 70 to 1200 mm/min during scanning. Moreover, a very critical parameter for the simulations was the value of the absorptivity coefficient [36], which was set to a mean value of n = 0.32 based on empirical measurements from tailored experiments that accounted for surface conditions and temperature-dependent effects. A sensitivity analysis has been conducted by varying the absorptivity coefficient by ±15% around the nominal value of 0.32. The results indicate that while absolute peak temperatures scale proportionally, the predicted transition thresholds between plastic deformation and melting, as well as the qualitative stress, strain and temperature distributions, remain unchanged. Within this range, groove depth predictions varied by less than ±10%.

The presented numerical framework enables detailed analysis of temperature fields, displacement, plastic strain, and stress evolution, providing deep insights into the thermomechanical response during laser surface processing.

## 3. Experimental Setup and Diagnostics

The experimental phase of this study was designed to generate robust data for the validation of the developed numerical models. This section details the integrated laser-CNC system, and the suite of diagnostic instruments employed to monitor the thermomechanical response of the material during laser processing.

### 3.1. Integrated Laser-CNC System

**Figure 2:**
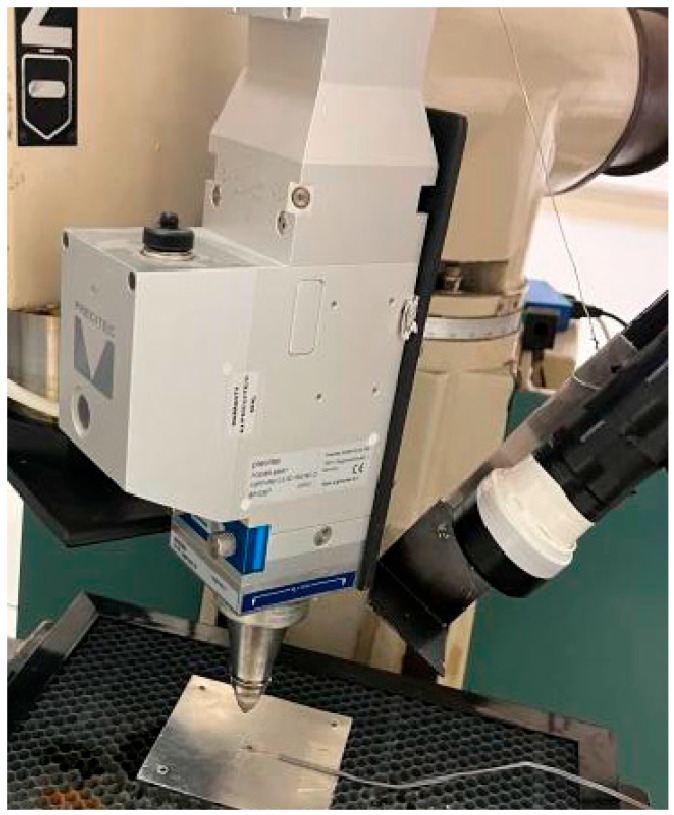
The cutting laser head mounted on the CNC and the Optris PI-1M thermal camera focused on the irradiated workpiece spot via the pinhole.

A high-power CW fiber laser system (TRUMPF TruFiber 2000 P compact, TRUMPF, Ditzingen, Germany) with a maximum output power of 2 kW and a wavelength of 1075 µm serves as the energy source. The laser beam is delivered through a Precitec LightCutter 2.0 (Precitec, Gaggenau, Germany) motorized cutting head, which is vertically mounted onto a Denford Easimill 3 (Denford, West Yorkshire, UK) 3-axis CNC milling machine via a custom-designed housing, as shown in Figure 2. This integration offers a versatile platform for both static and dynamic laser processing within a work area of 378 mm (X) × 180 mm (Y) × 80 mm (Z) with a positioning resolution of 0.01 mm. The laser cutting head features a focal length of 150 mm, producing a focused beam diameter of 250 µm at a working distance of 3 mm from the nozzle.

The laser and CNC operations are synchronized using a unified control software system. The Mach3 [44] software controls the CNC machine and the Trumpf Fiberview software (Version 3.7.1.0) [45] the laser parameters. A custom-designed PCB interface facilitates the communication of Mach3 over the Fiberview, ensuring precise triggering of the laser process cycles and laser power, at designated points along the toolpath.

The metal workpiece is 1 mm thick sheet of commercially pure aluminum alloy Al 1050 H14, selected for its uniform properties and well-documented thermomechanical characteristics. To prevent external mechanical constraints from influencing the results, the samples are freely positioned on a honeycomb metal supporting grid, rather than being clamped.

### 3.2. Experimental Diagnostics

A multi-diagnostic approach is employed to capture the thermal and mechanical response of the workpiece with high spatial and temporal resolution.

#### 3.2.1. Thermal Diagnostics

Thermal imaging system: To evaluate and validate the experimental and simulation results, a precise thermal diagnostic system is utilized. An Optris PI1M (https://optris.com/products/thermal-cameras/precision-line/pi-1m/ (accessed on 7 January 2026)) [46] thermal camera (f = 75 mm) is mounted on the CNC machine, focusing on the workpiece at 310 mm from the camera sensor, as presented in Figure 2 (left). The camera is equipped with a long pass filter with a cut-on wavelength of 750 nm to capture thermal data at a frame rate of 80 Hz. Additionally, two notch filters at the laser wavelength is placed in front of the camera to reduce laser beam reflections. The material emissivity is calibrated within the camera’s software and set to 0.05 for Al 1050. An iris diaphragm is employed to control the amount of light that reaches the camera, to prevent overexposure and ensuring accurate thermal measurements.

**Figure 3:**
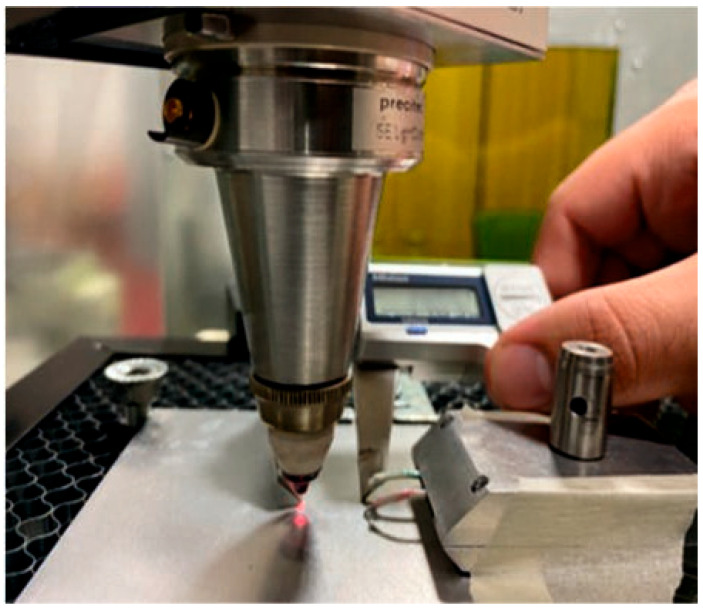
The workpiece positioned on the metallic honeycomb grid and the thermocouples mounted on the top surface of the Al workpiece.

Thermocouples: For localized temperature measurement, RS PRO K-type thermocouples (probe diameter: 0.3 mm) are positioned at precise distances (0.5–2 mm) from the laser irradiation spot. Their high response time (0.7 s) enables the tracking of transient thermal phenomena. Figure 3 depicts the thermocouples mounted on the top surface of the Al workpiece of the experiments.

#### 3.2.2. Mechanical and Surface Topography Diagnostics

White Light Interferometry (WLI): A non-contact WLI system, presented in [47,48], is used for post-process surface characterization. This technique utilizes the interference of light waves to reconstruct surface features with nanoscale precision. In WLI, white light—a broad-spectrum illumination source—is split into two beams: one reflects off the sample surface, while the other reflects off a reference mirror. These beams then recombine to create an interference pattern, which is analyzed to determine surface heights and features with exceptional accuracy. This optical technique provides high-resolution (nanometer-scale) measurements of surface topography, including the depth and width of the plastic deformed areas, as well as the etched or engraved areas.

Strain measurement system: To capture the mechanical response of the aluminum alloy workpiece to laser-induced thermal loading, a precise strain measurement system is implemented. This system utilizes strain gauges to measure surface deformation, supported by a custom electronic circuit for data acquisition and processing. The strain gauge used was the FLGB-02-11 from Tokyo Measuring Instruments (Tokyo, Japan) [49]. This gauge is constructed from a copper-nickel (Cu-Ni) alloy foil, making it suitable for measurements on heated materials, with a specified operational temperature range of up to 150 °C. The gauge is mounted on a 3.5 mm × 2.5 mm plastic carrier and was bonded to the metal surface using a high-temperature epoxy adhesive. The strain measurement system is based on a custom electronic setup developed around an Arduino Uno platform [50]. The operating principle relies on the fact that the strain gauge experiences a small change in electrical resistance (ΔR) when the material deforms. This change in resistance is measured using a quarter-bridge Wheatstone circuit, where one of the bridge resistors ($R_{4}$) is the active strain gauge. An HX711 24-bit analog-to-digital converter (AVIA (Avia Semiconductor), Fuzhou, China) is used to accurately measure the resulting imbalance voltage from the Wheatstone bridge. The Arduino processes the voltage data to calculate the strain in real-time, with results displayed on a computer via a custom data logging program. The strain (ε) is calculated from the output voltage ($V_{o}$) and the supply voltage ($V_{s}$) using the gauge factor (GF=2.07), as derived from the Wheatstone bridge equations. For a balanced bridge with identical resistors ($R_{1}=R_{2}=R_{3}=R_{gauge}$), the strain is given by the simplified formula:

(9) $$\varepsilon \approx \frac{4Vo}{Vs*GF}$$

**Figure 4:**
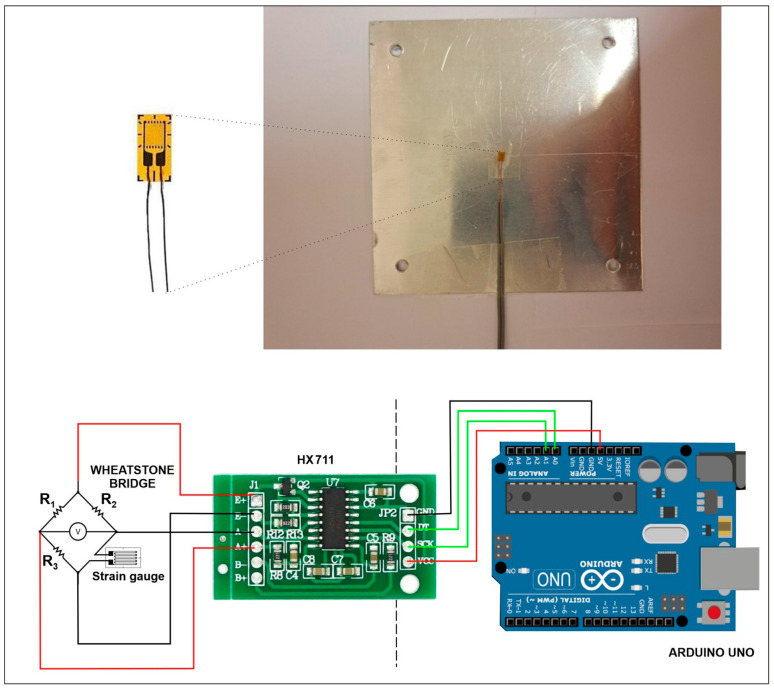
Strain gauge bonded onto the center of the specimen (**top**) and schematic diagram of the electronic setup for the strain measurements (**bottom**).

This system provides real-time, quantitative data on surface strain, which is critical for validating the mechanical response predicted by the FEM simulations. Figure 4 depicts the strain gauge and the electronic setup for the strain measurements.

## 4. Results and Discussion

This section presents a comprehensive evaluation of the thermomechanical response of Al 1050 H14 under static and dynamic CW fiber laser irradiation, combining high-fidelity FEM simulations with thermal, mechanical, and surface-topography diagnostics. Four representative case studies are examined, starting from non-ablative thermo-plastic deformation and progressing to the onset of etching and engraving. The results highlight the strong agreement between simulations and experiments across temperature evolution, plastic strain distribution, and final surface modification features. By systematically varying laser power and scanning speed, the analysis also reveals the transition mechanisms governing the shift from plastic deformation to material removal.

The effective absorptivity coefficient was determined independently from tailored preliminary experiments and constitutes the only calibrated model parameter. All other experimental measurements, including thermal-camera data, thermocouple readings, strain-gauge signals, and WLI surface profiles, were used exclusively for validation of numerical predictions.

### 4.1. Case Study: 200 W Static Beam—Plastic Effects

**Figure 5:**
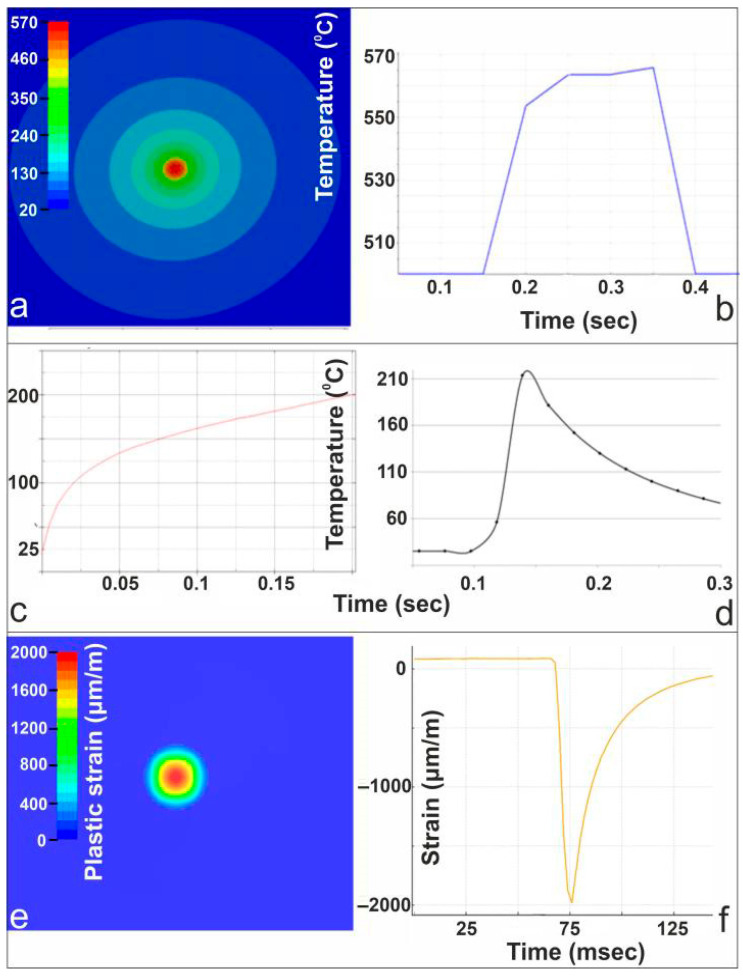
(**a**) FEM temperature field at 200 ms after laser irradiation; (**b**) thermal-camera temperature evolution; (**c**) FEM temperature evolution at 0.7 mm from the center of the irradiated spot; (**d**) thermocouple temperature measurement at 0.7 mm from the center of the irradiated spot; (**e**) FEM plastic strain field at 200 ms after laser irradiation; (**f**) strain-gauge total strain response during laser heating.

Figure 5 presents a combined numerical and experimental analysis of the thermomechanical response of Al 1050 under 200 W static laser irradiation. The FEM simulation in panel (a) shows the temperature distribution around the laser spot, with peak heating confined to the beam center, at 200 ms after laser irradiation. The corresponding thermal-camera measurement in panel (b), confirms the rapid rise to approximately 570 °C, which is below the melting point of Al 1050, during irradiation and subsequent cooling. Panel (c) provides the FEM-predicted transient temperature history at a point near the laser interaction zone, while the thermocouple data in panel (d) capture the sharp thermal transient experimentally. The measured thermocouple values are reconstructed based on a simplified thermal equilibrium model, as presented in [26]. The uncertainty associated with the reconstructed temperature arises mainly from the thermocouple tolerance, finite response time, and positioning relative to the surface; based on these factors, a conservative uncertainty of approximately ±5–10% is estimated for the reconstructed peak temperatures. At 0.7 mm from the center of the irradiated spot, the simulation predicted a maximum temperature of T = 205 °C, while for the same distance the thermocouple measured T = 215 °C, resulting in a percentage difference of 4.6%. The plastic strain field predicted by the FEM model is shown in panel (e), illustrating a localized region of permanent deformation beneath the beam center, at 200 ms after laser irradiation. Finally, panel (f) presents the strain-gauge measurement of total surface strain (elastic and plastic components), displaying a pronounced compressive excursion followed by partial recovery, consistent with the thermally induced mechanical response predicted by the simulation. Together, these results demonstrate strong agreement between the numerical model and the multi-diagnostic measurements. It should be noted that the strain gauges measure the total surface strain, which includes both elastic and plastic contributions, whereas the FEM results presented correspond to plastic strain only. Consequently, the comparison is qualitative and focuses on peak response magnitudes rather than direct one-to-one equivalence.

### 4.2. Case Study: 250 W Static Beam—Engraving Regime

When the local temperature exceeds the melting point, the mechanical response is governed by the strong temperature dependence of the Johnson–Cook model, leading to a substantial reduction in material stiffness and strength. Element erosion is activated once the accumulated damage parameter reaches unity and is interpreted here as a phenomenological representation of material separation under melt-softened conditions, rather than explicit modeling of melt flow or vaporization-driven removal.

**Figure 6:**
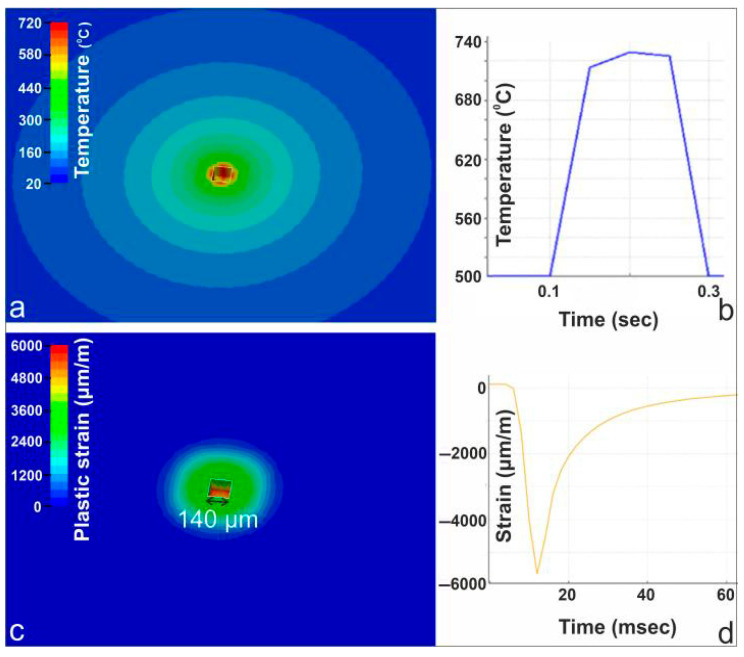
(**a**) FEM temperature field at 150 ms after laser irradiation; (**b**) thermal-camera temperature evolution; (**c**) FEM plastic strain distribution at 150 ms after laser irradiation; (**d**) strain-gauge response showing transient total strain during irradiation.

Figure 6 presents the combined numerical and experimental characterization of the 250 W static laser irradiation case. The FEM-generated temperature field in panel (a) shows a highly localized hotspot exceeding the melting point at the beam center, with smooth radial decay, at 150 ms after laser irradiation. The corresponding thermal-camera measurement in panel (b) peaks near 740 °C before cooling, in close agreement with the simulated thermal response. Panel (c) illustrates the FEM-predicted plastic strain distribution, revealing a compact plastically deformed zone, at 150 ms after laser irradiation. The material removed due to mechanical failure has a width of 140 µm and a depth of 100 µm. Panel (d) displays the strain-gauge response during irradiation, capturing a sharp compressive strain excursion approaching −6000 µm/m, followed by partial recovery during cooling. This strain evolution is consistent with the predicted concentration of plastic deformation at the beam center.

It should be noted that the present numerical framework captures phase change and material removal through an enthalpy-based thermo-mechanical formulation, allowing prediction of the onset and spatial extent of melting. However, detailed melt-pool hydrodynamics—including surface-tension-driven flow, recoil pressure effects, and melt ejection—are not explicitly resolved. As a result, the model is intended to predict the melt-affected and removed region geometry in a phenomenological sense rather than reproducing fine-scale melt-flow morphology. Consequently, validation against WLI measurements focuses on groove width, depth, and overall melt-affected extent rather than detailed surface flow features.

### 4.3. Case Study: 200 W Linear Moving Beam at 1200 mm/min—Plastic-Induced Surface Modification

**Figure 7:**
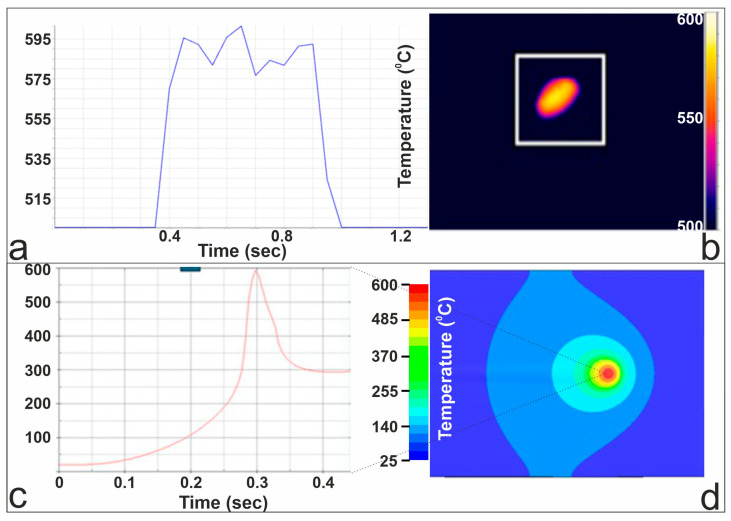
Dynamic irradiation at 1200 mm/min: (**a**) thermal-camera temperature evolution; (**b**) thermal image during laser scanning; (**c**) FEM transient temperature history; (**d**) FEM temperature field at 300 ms after the start of laser irradiation.

Figure 7 presents the thermal behavior of the aluminum sample during dynamic laser irradiation at a scanning speed of 1200 mm/min, comparing experimental measurements with FEM predictions. Panel (a) shows the thermal-camera temperature evolution recorded as the laser beam passes over the monitored region, where the temperature rapidly rises to approximately 595 °C before decreasing as the beam moves away. Panel (b) displays the corresponding thermal image, illustrating the elongated temperature footprint characteristic of a fast-moving heat source. In panel (c), the FEM-predicted transient temperature history at a point along the scan path is shown, capturing the sharp thermal peak produced by the brief laser exposure at this high scanning speed. At 0.7 mm from the center of the irradiated spot, 300 ms after the start of irradiation, the simulation predicted a maximum temperature of T = 600 °C. Panel (d) presents the FEM-computed temperature field, at 300 ms after the start of irradiation.

**Figure 8:**
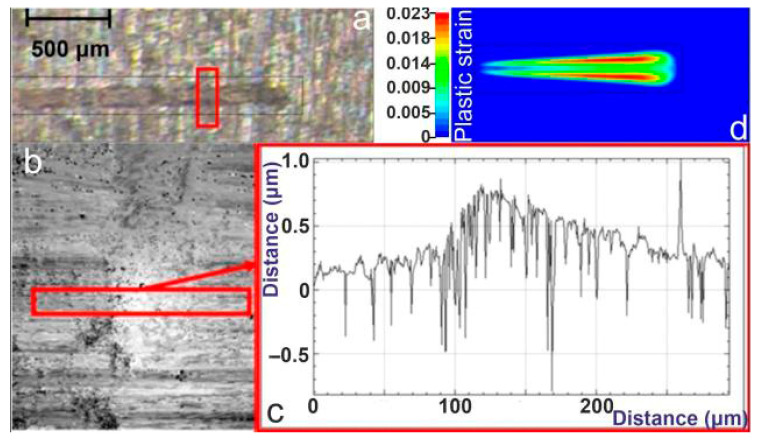
(**a**–**c**) WLI measurements of the laser-modified surface: top view, magnified region, and cross-sectional height profile; (**d**) FEM-predicted plastic strain distribution along the laser scanning path, at 300 ms after laser irradiation.

Figure 8 presents the experimentally measured surface profile obtained by WLI and the numerically predicted plastic strain distribution for the dynamic laser-irradiation case. Panel (a) shows the WLI top-view image of the processed track, where a narrow and shallow deformation region is visible along the laser path. A magnified WLI image in panel (b) highlights the elongated plastically affected zone, while panel (c) presents the corresponding cross-sectional height profile extracted along the marked region, showing surface deviations on the order of ±0.5 µm. These measurements confirm that the laser-induced deformation remains shallow and highly localized along the scan direction. Panel (d) displays the FEM-predicted plastic strain field, which exhibits a similarly elongated distribution with a decreasing plastic strain away from the beam path consistent with the geometry and extent of the experimentally observed deformation zone, at 300 ms after laser irradiation.

The strain distribution observed in the simulation exhibits a characteristic double-peak pattern, with two pronounced peaks flanking a smaller central one. This behavior is attributed to the moving thermal gradient and the associated evolution of the stress state during laser irradiation. The region directly beneath the laser beam undergoes rapid thermal softening, which promotes local stress relaxation, whereas adjacent regions experience stronger thermoelastic and plastic constraint during heating and subsequent contraction during cooling. As a result, compressive strain accumulates preferentially on either side of the instantaneous beam position rather than exactly at the centerline, leading to the observed double-peak strain signature.

### 4.4. Case Study: 200 W Linear Moving Beam at 75 mm/min—Onset of Etching

**Figure 9:**
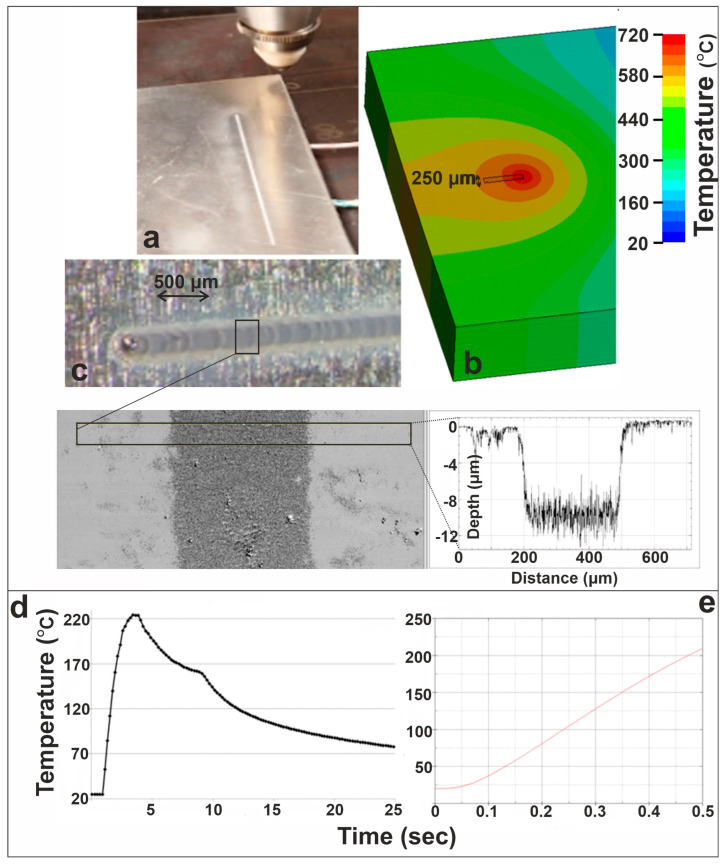
Case study: 200 W, linear beam, 75 mm/min. (**a**) Laser etching experiment; (**b**) FEM temperature field at 300 ms after laser irradiation; (**c**) WLI measurement and surface profile of etched groove; (**d**) thermocouple temperature evolution; and (**e**) FEM temperature evolution, at a fixed point 2 mm from the center of the laser irradiated spot.

Figure 9 presents the results of the 200 W linear laser irradiation at 75 mm/min, illustrating the onset of etching and validating the numerical predictions against experimental measurements. Panel (a) shows the laser etching experiment, where the CW beam produces a shallow etched track along the scan direction. The corresponding FEM temperature field in panel (b) indicates that the moving laser spot reaches temperatures high enough to initiate localized melting, forming an elongated high-temperature zone responsible for material removal, at 300 ms after the start of laser irradiation. WLI (panel (c)) confirms the formation of a well-defined etched groove, with the extracted surface profile revealing a depth of approximately 10 µm and a width of 250 μm. These measured values agree well with the simulated results that predicted the same values. Moreover, at a distance of 2 mm from the center of the moving laser beam, the K-type thermocouple positioned at this location measured a maximum temperature of 220 °C, while the FEM simulation predicted a maximum temperature of 210 °C at the same distance, resulting in a percentage difference of 4.5% at 500 ms after the onset of laser irradiation. Panel (d) shows the thermocouple measurements taken near the beam path, at a distance of 2 mm from the center of the moving laser spot, capturing the sharp temperature rise as the laser passes the closest point, followed by gradual cooling, while panel (e) illustrates FEM-predicted temperature evolution for the same distance near the beam path.

A direct comparison between the dynamic cases at 200 W, presented in Section 4.3 and Section 4.4, clearly demonstrates the dominant influence of scanning speed on the transition between thermo-plastic deformation and etching. The dwell time decreases from approximately 200 ms at 75 mm/min to 12 ms at 1200 mm/min (based on a 250 µm beam diameter), directly reducing the absorbed energy per unit length and preventing melt formation at higher speeds. At the higher speed of 1200 mm/min (Section 4.3), the beam interacts with each point on the surface for a significantly shorter time, resulting in lower effective heat accumulation. Consequently, although the peak temperatures approach ~600 °C, melting is not sustained, and only shallow, localized plastic deformation is observed—validated by both WLI measurements and FEM plastic-strain predictions.

In contrast, at the reduced speed of 75 mm/min (Section 4.4), the interaction time increases by more than an order of magnitude, allowing the temperature to exceed the melting threshold and remain elevated long enough for material removal to occur. This leads to a clearly defined etched groove, with depth (~10 µm) and width (~250 µm) matching the FEM predictions. The thermocouple and numerical temperature curves further highlight this distinction: while both cases exhibit sharp thermal transients, the slower-speed case shows sustained high-temperature exposure capable of initiating melt-driven etching. Thus, the comparison confirms that scanning speed functions as a critical control parameter, dictating whether the process remains in the thermo-plastic regime or transitions into material removal.

Looking ahead, the validated thermo-mechanical framework developed in this study provides a solid foundation for extending the numerical investigation toward full laser cutting simulations, where melt-flow dynamics, recoil pressure, keyhole formation, and material ejection play a dominant role. Unlike the deformation and moderate-etching regimes examined here, full cutting involves strongly coupled thermal–fluid–structural physics and complex free-surface evolution, making its modeling substantially challenging. Future work will therefore focus on integrating more advanced physical models—such as melt pool hydrodynamics, vaporization-driven recoil pressure, surface tension effects, and phase-change-driven material removal—to capture the key mechanisms governing kerf formation and cut quality.

## 5. Conclusions

This work presented a combined numerical and experimental study of the thermomechanical response of Al 1050 H14 under static and dynamic CW fiber laser irradiation. The developed 3D coupled thermo-mechanical FEM model accurately reproduced the temperature fields, plastic strain evolution, and final surface modifications across a range of laser powers and scanning speeds.

For the 200 W static case, the material response remained in the thermo-plastic regime, producing shallow permanent deformation in excellent agreement with multi-diagnostic measurements. At 250 W static irradiation, both simulations and experiments revealed localized melting and the onset of engraving, with the measured strain response closely matching the predicted strain evolution. In this context, the inherently high power of the CW fiber laser was effectively limited to a threshold value of 250 W by exploiting its modulated power control capability, allowing the investigation to remain within controlled surface-modification regimes.

Under dynamic irradiation, the interaction time was found to be a decisive factor. At a high scanning speed of 1200 mm/min, the surface experienced only brief thermal loading, leading to narrow and shallow plastic deformation without melting, a behavior captured with high fidelity by the FEM model. Reducing the scanning speed to 75 mm/min produced a clear transition to etching, as the longer exposure allowed temperatures to exceed the melting threshold and remain elevated long enough to induce material removal. This controlled adjustment of laser power and interaction time enabled systematic investigation of surface modifications limited to plastic deformation, etching, and shallow, high-precision engraving under both static single-shot and dynamic CW processing conditions. The agreement between measured and simulated temperature evolution, strain response, and surface geometry validates the predictive capability of the modeling framework for both plastic deformation and melting-dominated regimes.

Overall, the study demonstrates that the integration of high-resolution diagnostics with advanced FEM modeling provides a powerful tool for understanding and controlling laser–material interactions. The validated numerical framework offers a reliable means of optimizing laser parameters—power, dwell time, and scanning speed—to target specific outcomes ranging from plastic imprinting and marking to controlled etching and micromachining. The present model does not explicitly resolve melt-pool hydrodynamics, surface tension effects, or material redistribution. Instead, it focuses on predicting the onset of melting and material removal through a coupled thermo-mechanical framework. These additional physical mechanisms will be addressed in future work to extend the model toward full cutting and melt-flow-dominated regimes. The current work contributes new insight into CW laser processing of aluminum and establishes an investigation methodology suitable for industrial laser surface-modification applications.

## Figures and Tables

**Table 1:** Mesh Convergence Data.

Mesh Resolution (µm)	Peak Temperature (°C)	Deviation from Selected Mesh (%)
100 × 20 × 100	548	3.9
75 × 15 × 75	560	1.8
50 × 10 × 50 (selected)	570	-
25 × 10 × 25 (time expensive)	572	0.35

**Table 2:** Physical and elastic properties of Al 1050 (room temperature) [38,40].

Property	Symbol	Value	Unit
Density	ρ	2710	kg·m^−3^
Young’s modulus	E	68	GPa
Poisson’s ratio	ν	0.33	–
Bulk modulus	K	75	GPa
Shear modulus	G	26	GPa
Yield strength	YS	75	MPa
Ultimate tensile strength	UTS	100	MPa
Latent heat of melting	Lm	400	J/gr
Liquidus Temperature	LT	657	°C
Solidus Temperature	ST	646	°C

**Table 3:** Johnson–Cook plasticity and damage parameters [40].

Parameter	Symbol	Value	Unit
Initial yield stress	A	352	MPa
Hardening modulus	B	440	MPa
Strain-rate coefficient	C	0.0076	–
Strain hardening exponent	n	0.42	–
Thermal softening exponent	m	1.0	–
Reference temperature	T_0_	20	°C
Failure strain constant	D_1_	0.13	–
Pressure dependence	D_2_	0.13	–
Stress triaxiality exponent	D_3_	−1.5	–
Strain-rate dependence	D_4_	0.011	–
Temperature dependence	D_5_	1.0	–

**Table 4:** Temperature-dependent thermal properties of Al 1050 used in the FEM simulations [43].

Temperature (°C)	Thermal Conductivity (W·m^−1^·K^−1^)	Thermal Expansion Coef. (×10^−6^/K)	Specific Heat (J·kg^−1^·K^−1^)
20	230	24	900
100	235	25.4	930
200	230	26.6	980
300	220	27.8	1030
400	210	30.4	1080
500	200	32.9	1130
600	90	35.5	1200
800	100	55	1180
1000	110	60	1180
2000	145	71	1180
3000	165	-	1180

## Data Availability

The original contributions presented in this study are included in the article. Further inquiries can be directed to the corresponding author.
